# Correction: Gil-Almagro et al. Anxiety Evolution among Healthcare Workers—A Prospective Study Two Years after the Onset of the COVID-19 Pandemic Including Occupational and Psychoemotional Variables. *Medicina* 2024, *60*, 1230

**DOI:** 10.3390/medicina61020320

**Published:** 2025-02-12

**Authors:** Fernanda Gil-Almagro, Fernando José García-Hedrera, Cecilia Peñacoba-Puente, Francisco Javier Carmona-Monge

**Affiliations:** 1 Psychology Deparment, Universidad Rey Juan Carlos, Av. de Atenas, s/n, 28922 Madrid, Spain; fgilalmagro@gmail.com (F.G.-A.); cecilia.penacoba@urjc.es (C.P.-P.); 2 Nurse Intensive Care Unit, Hospital Universitario Fundación Alcorcón, Budapest, 1, 28922 Madrid, Spain; fjgarciah@gmail.com; 3 Anesthesia Department, Hospital Universitario Santiago de Compostela, Rúa da Choupana, s/n, 15706 A Coruña, Spain

In the original publication [1], there was a mistake in Table 4 as published. The unintentional error was made in Table 4 during data entry. Measurements for both time 2 and time 3 were entered as the same data. The corrected Table 4 appears below. The authors state that the scientific conclusions are unaffected. This correction was approved by the Academic Editor. The original publication has also been updated.

## Figures and Tables

**Table 4:** Anxiety level percentages at each of the time points of data collection.

		Time 1		Time 2		Time 3	
		n (%)	M (SD)	n (%)	M (SD)	n (%)	M (SD)
Anxiety			10.82 (5.86)		8.86 (5.41)		7.61 (5.13)
Grouped Anxiety	No anxi/Min ^2^	41 (16.0)		57 (22.2)		74 (28.8)	
	Mild	68 (26.5)		94 (36.6)		99 (38.5)	
	Moderate	78 (30.4)		65 (25.3)		64 (24.9)	
	Severe	70 (27.2)		41 (16.0)		20 (7.80)	
Anxiety Mode/Severe ^1^	Yes	148 (57.6)		106 (41.2)		84 (32.7)	

^1^ Anxiety Moderate/Severe ^2^ No anxiety/Minimal.
